# A Case Report of Acute Motor and Sensory Polyneuropathy as the Presenting Symptom of SARS-CoV-2

**DOI:** 10.5811/cpcem.2020.6.48683

**Published:** 2020-07-01

**Authors:** Michael R. Kopscik, Barbra K. Giourgas, Bradley C. Presley

**Affiliations:** * Medical University of South Carolina, Department of Emergency Medicine, Charleston, South Carolina; † Medical University of South Carolina, Department of Neurology, Charleston, South Carolina

**Keywords:** SARS-CoV-2, COVID, COVID-19, Miller Fisher syndrome, Guillain-Barré syndrome, motor and sensory polyneuropathy

## Abstract

**Introduction:**

Severe acute respiratory syndrome coronavirus 2 (SARS-CoV-2) typically presents with respiratory illness and fever, however some rare neurologic symptoms have been described as presenting complaints. We report a case of an acute motor and sensory polyneuropathy consistent with Miller-Fisher Syndrome (MFS) variant of Guillain Barre Syndrome (GBS) as the initial symptom.

**Case Report:**

A 31-year old Spanish speaking male presents with two months of progressive weakness, numbness, and difficult walking. He had multiple cranial nerve abnormalities, dysmetria, ataxia, and absent lower extremity reflexes. An extensive workup including infectious, autoimmune, paraneoplastic, metabolic and neurologic testing was performed. Initially SARS-CoV-2 was not suspected based on a lack of respiratory symptoms. However, workup revealed a positive SARS-CoV-2 polymerase chain reaction test as well as presence of Anti-Ganglioside – GQ1b (Anti-GQ1b) immunoglobulin G antibodies.

**Discussion:**

Miller Fisher syndrome (MFS) is a variant of Guillain-Barre syndrome (GBS) characterized by a triad of ophthalmoplegia, ataxia, and areflexia. The patient’s exam and workup including Anti-GQ1b is consistent with MFS.

**Conclusion:**

SARS-CoV-2 infection in patients can have atypical presentations similar to this neurologic presentation. Prompt recognition and diagnosis can minimize the risk of transmission to hospital staff and facilitate initiation of treatment.

## INTRODUCTION

Severe acute respiratory syndrome coronavirus 2 (SARS-CoV-2) typically presents with respiratory illness and fever; however, rare cases of isolated neurologic manifestations of this virus have been reported.^1^ Here we report a case of an acute motor and sensory polyneuropathy consistent with Miller Fisher syndrome (MFS) variant of Guillain-Barré syndrome (GBS) as the presenting symptom of SARS-CoV-2.

## CASE REPORT

A 31-year-old Spanish-speaking male with no significant past medical history presented to the emergency department (ED) with progressively worsening weakness, numbness, and difficulty walking. Approximately two months prior to presentation, he woke up and noticed some numbness in his right hand. One month later he noticed double vision. He started wearing an eye patch over one eye to help with symptoms of diplopia. He then started experiencing numbness in his right leg as well as left facial weakness with dysarthria. About a week later, his symptoms progressed to numbness on the left upper and lower extremity, which prompted a visit to an outside hospital. His workup was reportedly normal including a magnetic resonance imaging (MRI) of the brain and lumbar puncture. His weakness and bilateral paresthesias progressed to the point that he was unable to ambulate, which prompted his current visit. The patient denied any trauma, headache, neck stiffness, loss of vision, fever, cough, or any other infectious symptoms. He denied any recent travel, sick contacts, or contacts with similar complaints.

On presentation to the ED his vital signs were within normal limits, and with the exception of a malar facial rash and abnormal neurologic exam, his physical exam was unremarkable. On neurologic exam, he was awake, alert, and oriented appropriately. On primary gaze there was left-eye adduction and cranial nerve VI palsy bilaterally with extraocular movements and vertical nystagmus. He had a unilateral, lower motor neuron cranial nerve VII palsy with left upper and lower hemifacial weakness, and also cranial nerve XII dysfunction with tongue deviation to the left. Overall his motor bulk was normal and strength was 5/5 in flexor and extensor groups of all four extremities. His sensation was intact to light touch and pinprick in all extremities. He had significant dysmetria with finger-nose-finger and heel-to-shin bilaterally, slightly worse on the left with decreased amplitude and discoordination on finger tapping and other rapidly alternating movements bilaterally. All upper extremity reflexes were intact; however, he had no patellar or Achilles reflexes; he had downgoing plantar reflex on the left. and mute plantar reflex on the right. He was unable to stand or test gait secondary to significant ataxia.

The patient was admitted and underwent an extensive workup including infectious, autoimmune, paraneoplastic, metabolic, and neurologic testing. MRI of his brain and lumbar spine were unremarkable. A computed tomography of his chest looking for neoplasm revealed a consolidation in his left lower lobe that prompted SARS-CoV-2 polymerase chain reaction testing and returned positive. Cerebrospinal fluid (CSF) studies revealed the presence of anti-ganglioside – GQ1b (Anti-GQ1b) immunoglobulin G antibodies (1:100), with lymphocytic predominance without albuminocytologic dissociation, and he subsequently was found to have positive immunoglobulin G (IgG) antibodies to COVID–19.

The patient was treated with convalescent plasma, tocilizumab, and intravenous immunoglobulin, in addition to extensive physical and occupational therapy. He had some mild subjective improvement in vision and coordination as well as return of patellar reflexes bilaterally; however, he required maximum assistance to ambulate on transfer to rehab facility.

## DISCUSSION

MFS is a variant of GBS characterized by a triad of ophthalmoplegia, ataxia, and areflexia.^2^ It was first recognized as a distinct clinical entity in 1956 and is observed in 1–5% of GBS cases in Western countries.^2^,^3^ While GBS is characterized by ascending flaccid paralysis, symptoms of cranial nerve dysfunction predominate in MFS. The majority of MFS cases present following viral or bacterial infections, although it has also been reported in conjunction with autoimmune and neoplastic disorders as well. One widely-cited study reported a median of eight days between the onset of infectious symptoms and neurologic symptoms.^3^ Sensory, motor, and autonomic nerve dysfunction in MFS patients reflect immune-mediated nerve damage, likely due to molecular mimicry between viral/bacterial antigens and ganglioside GQ1b. Anti-GQ1b antibodies are present in about 90% of patients with MFS and are absent in normal subjects, making this an ideal diagnostic marker.^3^ The overall clinical picture must be considered for accurate diagnosis, as 26% of GBS patients and 66% of Bickerstaff’s brainstem encephalitis patients also test positive for these antibodies.

**CPC-EM Capsule**

What do we already know about this clinical entity?

Severe acute respiratory syndrome coronavirus 2 (SARS-CoV-2) is a novel coronavirus that has been found to have effects on multiple different systems throughout the body.

What makes this presentation of disease reportable?

Neurologic manifestations of SARS-CoV-2 have been reported, but this is the first case of acute motor and sensory polyneuropathy as the presenting complaint in emergency medicine literature.

What is the major learning point?

Maintaining a high suspicion and low threshold to test for SARS-CoV-2 is important to help minimize the spread of the disease.

How might this improve emergency medicine practice?

It is important to recognize the findings of neurologic syndromes and recognize their possible relationship to SARS-CoV-2.

This patient’s insidious onset of multiple cranial neuropathies, ataxia, and areflexia is suggestive of MFS, although his presentation was somewhat atypical. The presence of anti-GQ1b IgG antibodies (1:100) on CSF studies supports this diagnosis, despite the absence of expected albuminocytologic dissociation. Instead, lymphocytic predominance in this patient’s CSF suggests a sustained immune response to viral infection, SARS-CoV-2, within the CNS. This patient’s tongue fasciculations and generalized muscle atrophy are also a clinical finding not typically associated with MFS. Electromyography nerve conduction studies will be helpful in understanding this physiology. Angiotensin-converting enzyme 2 has been identified as a receptor for SARS-CoV-2, which is present throughout the nervous system, likely a contributing mechanism of this patient’s multiple neurologic manifestations.^4^

This patient lacked respiratory or infectious symptoms and presented exclusively with progressive neurologic deficits and an asymptomatic pulmonary infiltrate. In addition to the absence of expected symptoms, the extent of this patient’s neurologic deficits is atypical in a previously healthy patient infected with SARS-CoV-2.^1^,^5^,^6^ Because of his unique presentation, the patient was not tested for SARS-CoV-2 until hospital day 3, and later for antibodies as part of an extensive workup.

## CONCLUSION

Recognizing unique presentations of SARS-CoV-2 infection is especially pertinent for emergency physicians. This case highlights the importance of identifying SARS-CoV-2 infection in patients with atypical presentations, specifically multiple neurologic deficits, as this virus can exhibit multiple different neurologic manifestations. Prompt recognition and diagnosis of SARS-CoV-2 infection in the ED minimizes the risk of transmission to hospital staff and enables timely initiation of treatment to improve patient outcomes.
